# Response Consistency of Crowdsourced Web-Based Surveys on Type 1 Diabetes

**DOI:** 10.2196/43593

**Published:** 2023-08-18

**Authors:** Yu Kuei Lin, Sean Newman, John Piette

**Affiliations:** 1 Division of Metabolism, Endocrinology and Diabetes Department of Internal Medicine University of Michigan Medical School Ann Arbor, MI United States; 2 Department of Health Behavior and Health Education University of Michigan School of Public Health Ann Arbor, MI United States; 3 VA Ann Arbor Healthcare System Center for Clinical Management Research Ann Arbor, MI United States

**Keywords:** diabetes, Amazon Mechanical Turk, MTurk, crowdsourcing, survey, type 1 diabetes, T1D, cross-sectional survey, website, online platform, web-based platform, reliability

## Abstract

Although Amazon Mechanical Turk facilitates the quick surveying of a large sample from various demographic and socioeconomic backgrounds, it may not be an optimal platform for obtaining reliable diabetes-related information from the online type 1 diabetes population.

## Introduction

Patient registries for type 1 diabetes (T1D) are often developed through collaborations among large medical centers [1]. Amazon Mechanical Turk (MTurk), a confidential web-based crowdsourcing platform with more than half a million registered workers [2], may serve as an alternative route for cost-effectively surveying large samples of patients with T1D receiving care in geographically dispersed health care environments. In this study, we tested the feasibility of using MTurk to gather reliable information from people living with T1D.

## Methods

In April 2022, we conducted a cross-sectional survey with MTurk workers to evaluate the reliability of their survey responses about T1D using the consistency checks technique [3]. This study received institutional review board approval from the University of Michigan (HUM00212503). A step 1 screening survey was conducted to recruit people with a self-reported diagnosis of diabetes and to assess respondents’ sociodemographic information, health insurance type, and diabetes-related information (ie, type of diabetes, calendar year of diabetes diagnosis, types of health care providers seen for diabetes management, most recent hemoglobin A_1c_ level, and use of insulin and noninsulin diabetes medications). A compensation of US $0.50 was provided for completing this 2- to 3-minute survey. Respondents who reported having T1D in the screening survey were invited to complete the step 2 full survey, which included the same questions asked in the screening survey with additional questions derived from the T1D Exchange core questionnaire [1]. A compensation of US $3.30 was provided for completing this 15- to 20-minute survey. The workers’ IP addresses and geographical locations were also collected from the MTurk website. Per best practices for MTurk surveying, only workers with a high-quality task performance track record (ie, completing >1000 tasks; >90% of the completed tasks were approved by prior task requesters for payment [4-6]) were allowed to complete the surveys. All questions were set with force response to ensure a 100% response rate. Only US workers were eligible to participate in this study.

Response consistency was determined by comparing responses across the screening and full surveys. Predetermined criteria (ie, matching responses to questions in both surveys about biological sex, education level, insurance type, calendar year of diabetes diagnosis, and current insulin regimen) were set to identify eligible surveys for future research analysis. A descriptive analysis was conducted to calculate the rates of response consistency and eligible surveys.

## Results

**Figure 1:**
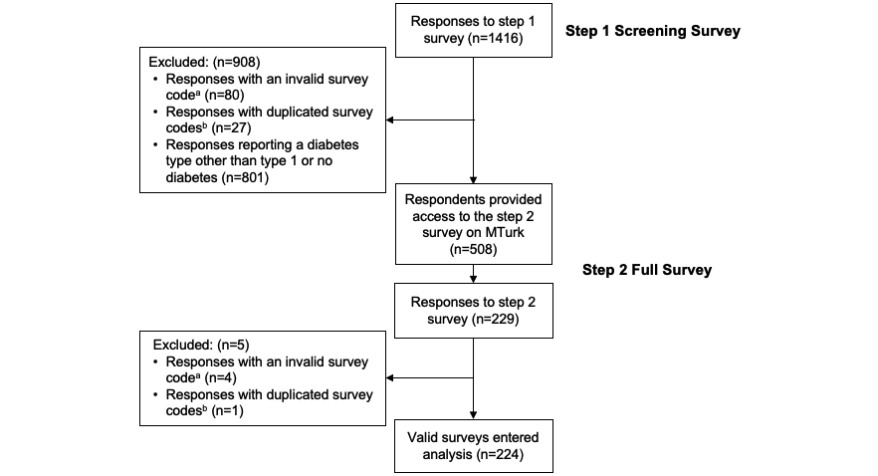
Participant flow chart. “a” indicates the respondent did not complete a survey and “b” indicates the respondent answered the survey more than once. MTurk: Amazon Mechanical Turk.

**Figure 2:**
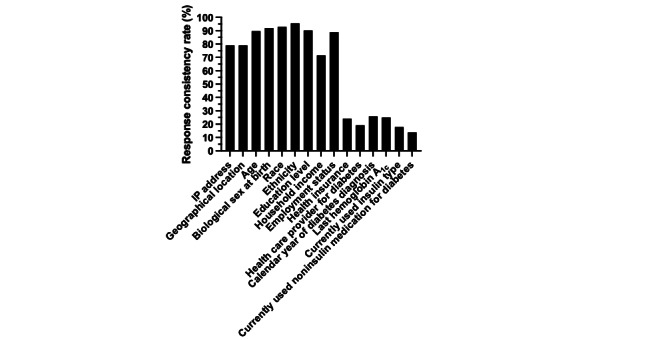
Screening and full survey response consistency rates.

A total of 1416 respondents completed the screening survey across 4 days. All 508 (36%) respondents who reported having T1D were invited to participate in the full survey, and 229 full surveys (45% of the initial T1D respondents) were completed within 3 days (ie, both surveys were completed within 1 week). After initial quality control, 224 surveys entered the analysis to determine response consistency (Figure 1). Comparing the screening and full surveys, more than 70% of the responses were identified as having the same MTurk IP address, geographical location, and demographic and socioeconomic information (Figure 2). In contrast, about 20% of respondents consistently reported health insurance or diabetes-related information in both surveys; for example, 26% (n=58) provided consistent responses about the calendar year of diabetes diagnosis. After applying the predetermined criteria for identifying eligible surveys, only about 6% (n=13) of the surveys were determined to be eligible for future research analysis.

## Discussion

Our findings suggest that identifying a population with T1D and gathering reliable information about their disease management through MTurk surveys could be challenging [7]. Despite screening a large number of patients reporting to have T1D, our study was unable to obtain a sufficient sample size of eligible surveys to generate meaningful data. These observations suggest that detailed assessments of patient-reported health conditions and outcomes through MTurk remain limited. However, MTurk could still serve as a strong platform for surveying the online population’s opinions and knowledge [8,9], given the high consistency rates in reporting demographic and socioeconomic information. A potential explanation could be the nature of the recruitment platform: although most MTurk workers may intend to genuinely perform tasks (as demonstrated by the high consistency rate in the sociodemographic information section), they also need to strike a balance between time and completing tasks quickly rather than accurately, as this cohort was recruited to “work” rather than to provide accurate information to make scientific contributions. Thus, if considering using MTurk to survey populations with specific medical conditions, simultaneous conduct of the survey in parallel with other platforms may help to determine the validity of findings from MTurk. Furthermore, prior research has demonstrated discrepancies in patient characteristics between cohorts recruited through MTurk and other platforms [10], and thus the generalizability of MTurk-based findings also remains to be further evaluated.

In conclusion, MTurk may not be an optimal platform for obtaining reliable responses about diabetes-related information from the online T1D population.

## Abbreviations

MTurk: Amazon Mechanical Turk
T1D: type 1 diabetes

## Data Availability

The data sets generated during and/or analyzed during this study are available from the corresponding author on reasonable request.
